# Evaluation of Flexural Behavior of Textile-Reinforced Mortar-Strengthened RC Beam Considering Strengthening Limit

**DOI:** 10.3390/ma14216473

**Published:** 2021-10-28

**Authors:** Jongho Park, Sun-Kyu Park, Sungnam Hong

**Affiliations:** 1 Global Frontiers of Resilient EcoSmart City, Sungkyunkwan University, Suwon 16419, Korea; rhapsode@skku.edu; 2 Department of Civil, Architectural and Environmental System Engineering, Sungkyunkwan University, Suwon 16419, Korea; skpark@skku.edu; 3 Department of Ocean Civil Engineering, Gyeongsang National University, Tongyeong 53064, Korea

**Keywords:** TRM, debonding, strengthening limit, TRM coefficient, flexural behavior

## Abstract

Textile-reinforced mortar (TRM) is a strengthening material in which textiles are attached to reinforced concrete (RC) structures using an inorganic matrix. Although many studies on structural behavior, various factors that affect TRM behavior could not be determined clearly. Especially, the uncertainty in bonds due to inorganic materials was not considered. In this study, the flexural behavior of TRM-strengthened beams was determined considering intermediate crack debonding occurred. The TRM beam strengthening limit and TRM coefficients were defined considering the possibility of premature failure and experimental results of four other research on 22 specimens. Therefore, it is expected that a conservative design would be possible when the suggested strengthening limit coefficient is applied.

## 1. Introduction

Various materials and methods exist for strengthening reinforced concrete (RC) structures. Fiber-reinforced polymer (FRP) has been the most commonly studied material for external reinforcement for about 30 years, and FRP external bonding is currently the most widely applied strengthening method. FRP has advantages such as high strength, high strength/weight ratio, high resistance to fatigue and corrosion, and ease of construction. However, it cannot be used on wet surfaces because of the use of organic materials such as epoxy resin. It also has disadvantages such as low glass transition temperature, low fire resistance, and water permeability [1,2].

To overcome the disadvantages of using organic materials and maintain the mechanical characteristics of fiber reinforcement, which is advantageous for structural reinforcement, the strengthening method with textile-reinforced mortar (TRM) using inorganic materials (hereinafter referred to as matrix) has been actively studied [3,4,5,6,7]. Textile is a strengthening material in which fibers, such as carbon, glass, and aramid, are woven in two or more directions. The fibers (yarn or roving) are made of thousands of filaments. TRM is a structural strengthening material in which textiles, composed of fibers with excellent tensile strength and chemical resistance, are attached to the surface of masonry and RC structures using a matrix such as cement mortar. Efficient construction is possible in various environments by using a matrix because the inorganic material is resistant to temperature changes and can be used on wet surfaces.

Various studies have been conducted to analyze the structural behavior of TRM-strengthened RC beams (TRM beams). Textiles are densely packed with thousands of fibers; thus, cement particles do not easily penetrate the textile. Therefore, the bond between the textile and matrix is not uniformly perfect as only the outer fibers are bonded [8,9,10,11]. The bond behavior exhibited by the inorganic matrices is relatively uncertain compared to that of organic materials. Therefore, the bond behavior of the TRM and concrete substrate was determined through a direct shear and beam test using the assumed local bond stress-sliding model, FRP bond strength evaluation model, and various design factors considering structural and environmental condition [12,13,14,15,16,17,18].

Several experimental studies have been conducted on the flexural behavior of TRM beams considering variables such as textile reinforcement ratio, textile configuration, steel reinforcement ratio, matrix type, textile shape, and textile anchorage. TRM beam behavior has been evaluated and efficient strengthening methods for textiles have been developed [2,19,20,21,22,23]. However, fiber slips have been reported because of inefficient bonding between the textile and matrix. To solve this problem, a study was conducted to investigate textiles reinforced by impregnation or coating with organic materials such as epoxy [24,25,26]. Studies have been conducted to prevent local textile bending and maintain accurate location at the designed reinforced axis by fixing both ends of the textile and manual stretching [27,28,29,30]; these studies had limitations because a constant tensile force was not introduced. In addition, to maximize the textile performance, studies on textile-reinforced concrete (TRC) and TRM using the pre-tension method have been conducted [31,32,33,34].

Various factors affect the performance and flexural behavior of the TRM, and it is difficult to evaluate or design the TRM considering all individual factors [35]. ACI Committee 549 [36] proposed an effective tensile strain through a uniaxial tensile test for TRM reinforcements and suggested a method for evaluating the flexural strength of TRM beams. Alrshoudi [37] and Kamani et al. [30] considered effective factors for the textile to reflect incomplete textile and matrix bonds in the flexural strength evaluation. Ombres et al. [21] and Raoof et al. [26] determined the failure behavior and flexural strength by evaluating the bond strength between the TRM reinforcement and concrete substrate. In addition, the flexural behavior was evaluated through a sectional analysis based on the strain compatibility and force equilibrium conditions [25]. A wide variety of flexural behavior evaluation methods for TRM beams are presented according to their design factors, but they assume a perfect bond between the TRM reinforcement and concrete substrate until the ultimate stage. These assumptions cannot consider the uncertainty in bonds due to the use of inorganic materials and do not reflect the behavior of TRM beams under service load [27,34].

This study proposes a method for evaluating the flexural behavior of TRM beams considering premature failure related to bond uncertainty and attempts to determine under service load. A strengthening limit was proposed by comprehensively considering various factors affecting the TRM strengthening method based on the experimental results of Park et al. [34].

## 2. Strengthening Limit of TRM

The TRM exhibits a bilinear stress–strain relationship owing to the composite behavior of the textile and matrix [36]. In addition, the bonding problem between the textile and matrix, and TRM and substrate of RC is always manifested in the TRM beam. Therefore, the bond characteristics must be considered in the evaluation of the flexural behavior. In the case of FRP, strengthening design considers the FRP delamination as the main failure mode and limits the effective strain in the FRP at a value for which debonding may occur [38]. However, the failure modes in the TRM beam are varied compared to the FRP-strengthened RC beam, and the criteria for evaluating the flexural strength considering the uncertainty are insufficient. Therefore, to conservatively evaluate the flexural behavior of TRM beams, the concept of the strengthening limit was applied. Strengthening limits should be derived by comprehensively considering various factors, and this study considers the possibility of premature failure under service load and the experimental results by Park et al. [34].

## 3. Experimental Program

In a recent study, Park et al. [34] used pure textiles that did not undergo an impregnation or coating process and analyzed the effect of textile straightening to prevent local bending inside the matrix. The experimental design and results presented by Park et al. [34] are summarized.

### 3.1. Material

Alkali resistant (AR)-glass and carbon textile were used in the experiment, and the textile properties are listed in Table 1. The AR-glass textile had an interval of 8 mm × 8 mm, and both warp and weft roving were fixed by extra filaments along the warp direction. The carbon textile had an interval of 10 mm × 10 mm, and both roving directions were fixed using thermal bonded filaments. The textile was cut to fit the 120 mm width of the RC so that the AR-glass textile had 12 warps and the carbon textile had 6 warps roving.

The concrete used for the RC beam was ready-mixed concrete with a specified concrete strength of 35 MPa, and a polymer mortar with a specified strength of 45 MPa was used. Table 2 and Table 3 show the average compressive strength of the matrices and the flexural and bond strengths of the polymer mortar, respectively. The compressive strength was measured using 150 mm × 300 mm cylindrical specimen. The mix proportion of both matrix has been described in detail in previous study by author [34].

### 3.2. Experimental Set-Up

The main experimental variables included textile type and textile reinforcement ratio, and straightening was considered. Approximately 20% (denoted as Lo1), 60% (denoted as Lo2), and 130% (denoted as O) of the balanced reinforcement ratio, calculated according to ACI 549.4R-13 [36], were applied as the textile reinforcement ratios. A tensile force equivalent to 5% of the tensile strength of the filament was applied to prevent local bending and straighten the textile. Detailed experimental parameters are listed in Table 4. AR and Ca denote AR-glass and carbon textiles, Lo denotes low-reinforced textile, O denotes over-reinforced textile, and S denotes straightened textile.

**Figure 1:**
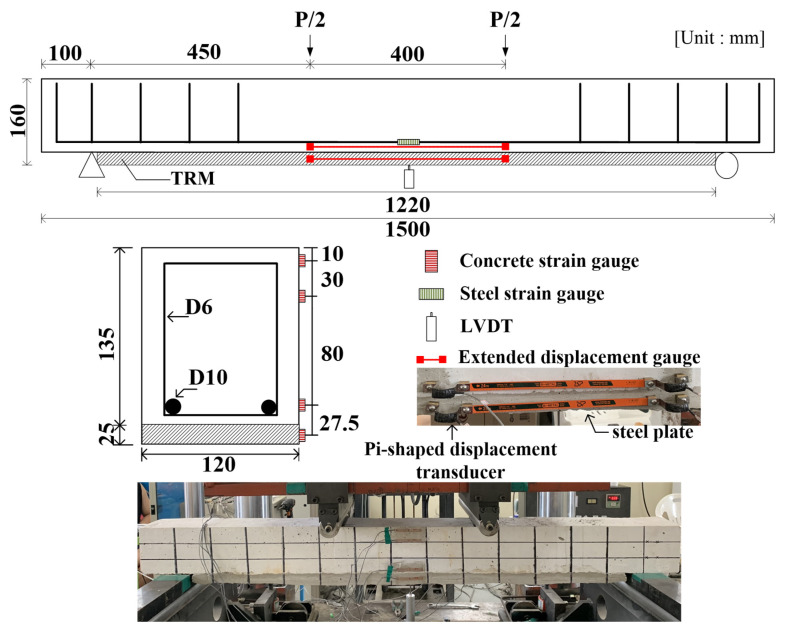
Schematic of specimen and test setup.

A schematic of the specimen and test setup is shown in Figure 1. Steel reinforcement with a diameter of 9.53 mm was used for tensile reinforcement, and a diameter of 6.35 mm was used for the stirrup. The yield strength of each steel reinforcement used in the experiment was 400 MPa, and the elasticity modulus was 200 GPa. The specimen span was 1300 mm, and the TRM reinforcement was 1220 mm, excluding the length of the supporting point. The substrate of the RC beam was ground with a grid of grooves before pouring the primer and polymer mortar. The textile and mortar were repeatedly applied up to the design level. The thickness of TRM was 25 mm, which slightly higher than conventional 1 cm value, to provide sufficient mortar thickness for bonding between mortar and textile. In the case of straightening, each textile was straightened and fixed by the clamp device and steel plate with anchor, and straightening force was measured by S-Beam-shaped loadcell. The detailed strengthening process was described in detail previous study by author [34]. Four points were loaded using a 2000 kN universal testing machine (UTM), and the displacement was controlled at a speed of 0.1 mm/s.

### 3.3. Experimental Results

#### 3.3.1. Failure Mode

A flexural crack was first created at the center of the beam. After the steel reinforcement yielded, textile slippage in the crack increased significantly, immediately followed by intermediate crack debonding of the TRM reinforcement and textile rupture. Subsequently, a compressive failure occurred. The failure modes in the specimen are summarized in Table 5 and were arranged in the order observed after the steel reinforcement yielded.

**Figure 2:**
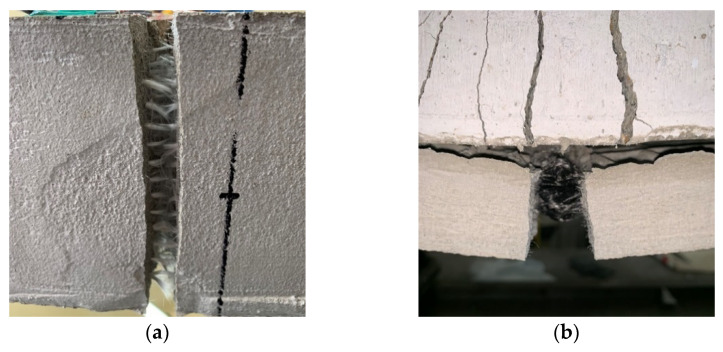
Failure modes: (**a**) Textile rupture; (**b**) IC debonding.

Similar failure modes were observed in all specimens except CaLo2, which was dominated by the textile slippage behavior. In the specimens without textile straightening, textile ruptures were observed, and in the specimens in which straightening was introduced, intermediate crack debonding (referred to as IC debonding) failure of the TRM reinforcement was observed. IC debonding is a phenomenon in which the TRM reinforcement does not have infinite strains across the flexural crack in the maximum moment section, and thereby, horizontal cracks propagate toward the nearer end of the plate [39,40]. Textile straightening can improve the tensile strain capacity of the TRM plate; therefore, IC debonding is the main reason for the significant improvement in the load resistance capacity of the TRM beam through textile straightening. Figure 2 shows the textile rupture and IC debonding induced by the intermediate cracking.

#### 3.3.2. Load and Deflection

**Figure 3:**
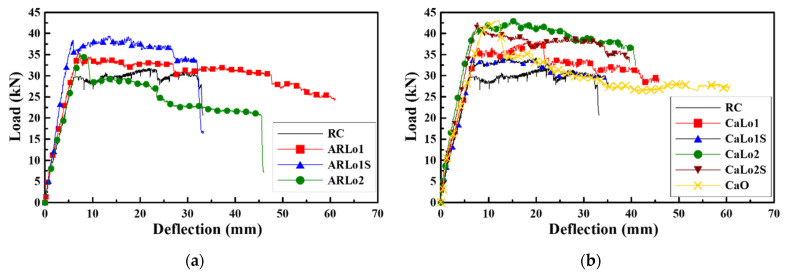
Load and deflection curves: (**a**) AR-glass TRM; (**b**) Carbon TRM.

Table 6 and Figure 3 present the load-deflection results. The crack point was assumed where the value of the crack gauge changed rapidly, and the service load stage was determined to meet all the serviceability limit criteria of ACI 440.2R-17 [38], as shown in Equations (1) and (2). The yield stage was based on the steel reinforcement yield point. The ultimate stage was the section when compression cracks begin to develop, at which point the maximum load is achieved.

(1) $$f_{s,s}\leq 0.80f_{y}$$

(2) $$f_{c,s}\leq 0.60f_{ck}$$

where $f_{s,s}$ is the stress in the steel reinforcement under service load, $f_{c,s}$ is the compressive stress in concrete under service load, $f_{y}$ is the steel yield strength and $f_{ck}$ is concrete compressive strength.

In the service load stage, compared to the RC specimen, the load increased in all the specimens except in ARLo2 and CaLo1S; however, the increase was less than that in the yield stage. The yield load of the TRM beam increased compared to that of the RC specimen, but the load could not be resisted sufficiently owing to the failure of the TRM reinforcement. Using AR-glass textile, the TRM beam loads were on average 12%, 23%, and 24% larger in the service, yield, and ultimate stages, respectively, compared to those of the RC specimen. Using carbon textile, the TRM beam loads were on average 8%, 32%, and 30% larger in the service, yield, and ultimate stages, respectively, compared to those of the RC specimen. The strengthening efficiency of TRM beam was maximized at the yield stage. An average of 22% larger deflection was observed due to an increase in load at the yield stage, but it was confirmed that there was only a slight increase in the overall flexural stiffness.

## 4. Analysis and Discussion

### 4.1. Prediction of Flexural Behavior Considering Strengthening Limit

Escrig et al. [23] suggested that TRM beams are more efficient at the yield stage than at the ultimate stage. The reason for this was textile slippage, damage, and rupture as the load increased due to friction with the matrix. Based on the results presented in Section 3, it was revealed that the TRM beam showed high strengthening efficiency up to the yield stage, and the composite behavior, possibly terminated following the TRM reinforcement failure. Therefore, the steel reinforcement yielding point is assumed to be the strengthening limit at which the TRM reinforcement performance is maximum.

**Figure 4:**
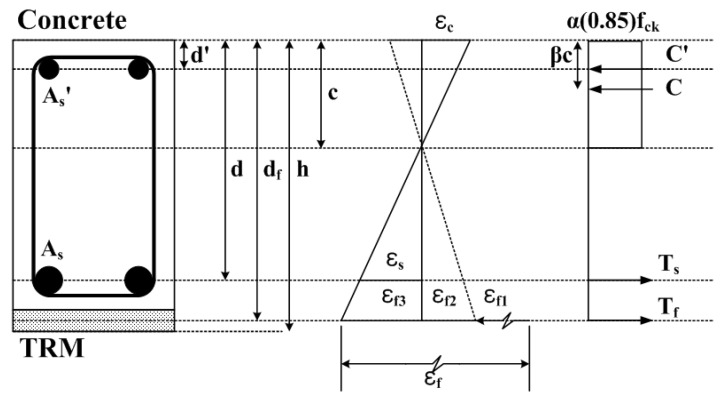
Strain, stress distribution, and internal forces of TRM beam.

To predict the flexural behavior, the flexure theory considering the strain compatibility and force equilibrium condition of the cross-section was presented. Figure 4 shows the strain, stress distribution and internal forces of the TRM beam cross-section in the strengthening limit. Deflection was predicted using the unit load method based on the moment–curvature relationship. The basic assumptions, which are considered valid up to the strengthening limit of the TRM beam, for predicting the flexural behavior of TRM beams are as follows:

- Plane sections remain plane after loading;
- After cracking, the tensile strength of the concrete and polymer mortar was neglected;
- At the same location, the strains in concrete, steel, and TRM reinforcement are the same;
- Textiles resist longitudinal loads only, ignoring the effects of transverse fibers.

#### 4.1.1. Uncracked Stage

The TRM beam in the uncracked stage exhibits linear elastic behavior until the tensile stress on the tensile side reaches the tensile strength of the concrete. A tensile force can be introduced into the textile to prevent local bending, and the cracking moment and curvature considering the tensile force can be obtained from the tensile strength of concrete, as shown in Equations (3) and (4).

(3) $$M_{cr}=\left(f_{r}+\frac{P_{f}}{A_{g}}+\frac{P_{f}e_{f}}{I_{g}}y_{b}\right)\frac{I_{g}}{y_{b}}$$

(4) $$\kappa_{cr}=M_{cr}/\left(E_{c}I_{g}\right)$$

where $f_{r}$ is the tensile strength of concrete ($=0.3{\left(f_{cm}\right)}^{2/3}$), $P_{f}$ is the tensile force for textile straightening, $e_{f}$ is the eccentricity distance of the textile, $A_{g}$ and $I_{g}$ are the total area and gross moment of inertia of the TRM beam, respectively, $y_{b}$ is the distance from the neutral axis to the bottom of the TRM beam, $E_{c}$ is the concrete elasticity modulus, and $f_{cm}$ is the mean compressive strength of concrete ($=f_{ck}+\Delta f$).

#### 4.1.2. Service Load Stage

In the service load stage (cracked stage), the tensile load is entirely handled by the steel and TRM reinforcement, and the tensile strength of the concrete is ignored. The flexural strength was predicted based on the serviceability limit, as shown in Equations (1) and (2).

If the stress distribution in the concrete is converted to an equivalent rectangular stress block by assuming that the strain at one edge is 0 and the effective stress in the concrete is 0.85, the concrete stress block factors *α* and *β* can be obtained as shown in Equations (5) and (6) [41].

(5) $$\alpha =\frac{10^{3}\varepsilon_{c}}{2}-\frac{{\left(10^{3}\varepsilon_{c}\right)}^{2}}{12},\;\beta =\frac{8-10^{3}\varepsilon_{c}}{4\left(6-10^{3}\varepsilon_{c}\right)},\;0\leq \varepsilon_{c}\leq 0.002$$

(6) $$\alpha =1-\frac{2}{3\left(10^{3}\varepsilon_{c}\right)},\;\beta =\frac{4\left(10^{3}\varepsilon_{c}\right)-3{\left(10^{3}\varepsilon_{c}\right)}^{2}-2}{6\left(10^{3}\varepsilon_{c}\right)\left(0.67-10^{3}\varepsilon_{c}\right)},\;0.002\leq \varepsilon_{c}\leq 0.0033$$

The compressive strain $\varepsilon_{c}$ of the concrete and strain $\varepsilon_{c}^{\prime }$ of the compression steel reinforcement are expressed as follows:

(7) $$\varepsilon_{c}=c_{s}\varepsilon_{s,s}/\left(d-c_{s}\right)$$

(8) $$\varepsilon_{s}^{\prime }=\left(c_{s}-d^{\prime }\right)\varepsilon_{s,s}/\left(d-c_{s}\right)$$

where $c_{s}$ is the neutral axis depth at the service load stage, d and $d^{\prime }$ are the effective depths of the tensile and compression steel reinforcements, respectively, and $\varepsilon_{s,s}$ is the steel strain based on the serviceability limit.

The total strain $\varepsilon_{f}$ in the textile, which occurs during the TRM reinforcement, is equal to the sum of strains $\varepsilon_{f1}$, $\varepsilon_{f2}$, and $\varepsilon_{f3}$.

(9) $$\varepsilon_{f1}=P_{f}/\left(A_{f}E_{f}\right)$$

(10) $$\varepsilon_{f2}=\left(P_{f}/E_{c}I_{g}\right)\left(I_{g}/A_{g}+e_{f}^{2}\right)$$

(11) $$\varepsilon_{f3}=\left(d_{f}-c_{s}\right)\varepsilon_{s,s}/\left(d-c_{s}\right)$$

where $\varepsilon_{f1}$ is the strain in the textile due to the tensile force for straightening, $\varepsilon_{f2}$ is the strain that occurs in the textile during decompression, when the textile strain becomes zero, $\varepsilon_{f3}$ is the strain occurring in the textile until the steel reinforcement yields as the load increases after decompression, $A_{f}$ is the area of textile reinforcement, $E_{f}$ is the textile elasticity modulus, and $d_{f}$ is the effective textile depth.

For the service load stage, the TRM beam force equilibrium condition is expressed as Equation (12), and the flexural strength can be calculated from Equation (13) using the moment equilibrium condition.

(12) $$\alpha \left(0.85f_{ck}\right)bc_{s}+A_{s}^{\prime }f_{s}^{\prime }-A_{s}f_{s}-A_{f}f_{f}=0$$

(13) $$M_{s}=A_{s}f_{s}\left(d-\beta c_{s}\right)+A_{f}f_{f}\left(d_{f}-\beta c_{s}\right)-A_{s}^{\prime }f_{s}^{\prime }\left(\beta c_{s}-d^{\prime }\right)$$

where b is the width of beam, $A_{s}^{\prime }$, $A_{s}$ and $A_{f}$ are the area of compressive steel reinforcement, the tensile steel reinforcement and textile reinforcement, respectively, and $f_{s}^{\prime }$, $f_{s}$ and $f_{f}$ are the stress of the compressive steel reinforcement, tensile steel reinforcement and textile reinforcement.

#### 4.1.3. Yield Stage

The yield stage occurs when the tensile steel reinforcement strain reaches the yield strain $\varepsilon_{y}$.

(14) $$\varepsilon_{c}=c_{y}\varepsilon_{y}/\left(d-c_{y}\right)$$

(15) $$\varepsilon_{s}^{\prime }=\left(c_{y}-d^{\prime }\right)\varepsilon_{y}/\left(d-c_{y}\right)$$

(16) $$\varepsilon_{f3}=\left(d_{f}-c_{y}\right)\varepsilon_{y}/\left(d-c_{y}\right)$$

For the yield stage, the force equilibrium condition of the TRM beam is given by Equation (17), and the flexural strength can be calculated from Equation (18) using the moment equilibrium condition. The curvature at the yield stage is given by Equation (19).

(17) $$\alpha \left(0.85f_{ck}\right)bc_{y}+A_{s}^{\prime }f_{s}^{\prime }-A_{s}f_{y}-A_{f}f_{f}=0$$

(18) $$M_{y}=A_{s}f_{y}\left(d-\beta c_{y}\right)+A_{f}f_{f}\left(d_{f}-\beta c_{y}\right)-A_{s}^{\prime }f_{s}^{\prime }\left(\beta c_{y}-d^{\prime }\right)$$

(19) $$\kappa_{y}=\varepsilon_{y}/\left(d-c_{y}\right)$$

#### 4.1.4. Deflection

It is necessary to predict the deflection at the service load stage for serviceability verification, and it is calculated using the effective moment of inertia. The curvature $\kappa_{a}$ for an arbitrary moment $M_{a}$ and the effective moment of inertia $I_{e}$ can be obtained from Equations (20) and (21), respectively, using the tri-linear moment–curvature relationship. The TRM beam is assumed to be an elastic body in the service load stage.

(20) $$\kappa_{a}=\kappa_{cr}+\left(M_{a}-M_{cr}\right)\left(\kappa_{y}-\kappa_{cr}\right)/\left(M_{y}-M_{cr}\right)$$

(21) $$I_{e}=M_{a}/\left(\kappa_{a}E_{c}\right)$$

Therefore, the deflection δ of the TRM beam under an arbitrary load can be obtained from Equations (22) and (23) using the moment area and unit load method.

(22) $$\delta =2\times \left(\int_{0}^{a}\frac{mM_{1}}{E_{c}I}d_{x}+\int_{a}^{L/2}\frac{mM_{2}}{E_{c}I}d_{x}\right)$$

(23) $$\delta =\left(P/2\right)\left(3L^{2}-4a^{2}\right)a/\left(24E_{c}I\right)$$

where a is the distance from the support to the loading point, L is the span length, m is the moment by unit load, $M_{1}$ is the moment in the distance a, $M_{2}$ is the moment between loading point, and I is the effective moment of inertia for an arbitrary load.

### 4.2. Comparison

The properties required for predicting the flexural behavior of the TRM beams were applied according to the experimental results. For the matrix, the same properties as those of concrete were used because the ratio of the elasticity modulus of the concrete to that of the polymer mortar is 1.05, which results in a negligible change in the moment of inertia. The steel reinforcement yield strain in the RC specimen was 2500 μ (mm/mm), and the average yield strain in the TRM beam was 2900 μ (mm/mm).

Table 7 lists the experimental and predicted results. For the uncracked stage, the load and deflection test results of the TRM beam were on average 43% smaller and 59% larger, respectively, than the predicted results. The loads at the service load and yield stages were on average 9% and 10% smaller, respectively, than the predicted results. During the service load and yield stages, textile slippage occurred because the matrix and textile could not be integrated, and there was a sign of TRM reinforcement debonding. The prediction results of specimens ARLo1S and CaLo2S with textile straightening showed high accuracy. The experimental results of deflection at the service load and yield stages were on average 16% and 15% larger, respectively, than the predicted results. This is because the initial flexural stiffness was lower than that in the ordinary state owing to the specimen design and construction, assuming the damage.

From the comparison it was observed that the predicted flexural behavior of the TRM beam, using the strain compatibility and force equilibrium conditions, showed a trend similar to the experimental result, but it was not predicted conservatively.

### 4.3. Proposal of Conservative Evaluation of TRM Beam

Various failure factors, such as textile slippage, damage, and debonding of the TRM reinforcement, made it difficult to predict the exact flexural behavior of the TRM beam. However, despite the presence of various TRM reinforcement failure factors, the TRM beam behavior was relatively stable, and sufficient strengthening efficiency was observed in the strengthening limit section. Conservative prediction and designing of the flexural behavior were not possible because of the factors causing premature failure. Therefore, based on the strengthening limit of the TRM beam, the coefficient for conservative design was presented, considering the results of previous studies by other researchers. Because it is difficult to consider all the individual factors that may affect the flexural behavior of TRM beams, only the final experimental results were considered.

Table 8 presents specimens with similar experimental conditions and debonding. Park et al. [27] presented experimental results similar to Park et al. [34]. The results of the study by Ombres [13,21] indicated that TRM reinforcement IC debonding occurred; however, the load showed a tendency to increase continuously.

For a conservative design of flexural strength, the flexural strength coefficient ($\phi_{TRM}$) of the TRM beam was assumed to have a 95% probability from the standard normal distribution and could be expressed as 0.99 − 1.64 × 0.11 = 0.81, as shown in Equation (24).

(24) $$M_{TRM}=\phi_{TRM}M=0.81\left\{A_{s}f_{s}\left(d-\beta c\right)+A_{f}f_{f}\left(d_{f}-\beta c\right)-A_{s}^{\prime }f_{s}^{\prime }\left(\beta c-d^{\prime }\right)\right\}$$

The coefficient for TRM beam deflection was applied to the effective moment of inertia instead of the value of deflection, and the 10% trimmed mean value was used considering the high deviation between the experimental and predicted results. The mean, standard deviation, and variance of the effective moment of inertia were 0.88, 0.17, and 0.028, respectively. Therefore, the deflection coefficient ($\lambda_{TRM}$) of the TRM beam was assumed to have a 90% probability from the standard normal distribution, and it could be expressed as 0.88 − 1.29 × 0.17 = 0.66, as shown in Equation (25).

(25) $$I_{e,TRM}=\lambda_{TRM}I_{e}=0.66M_{a}/\left(\kappa_{a}E_{c}\right)$$

**Figure 5:**
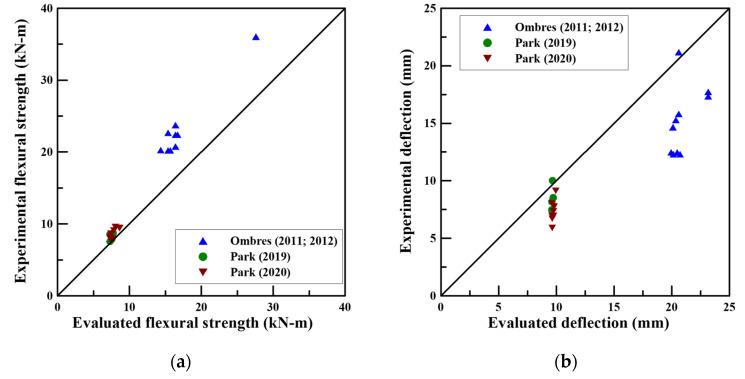
Comparison of experimental and evaluated results: (**a**) Flexural strength; (**b**) Deflection.

Figure 5 shows the flexural strength and deflection results obtained by introducing a strengthening limit coefficient. For flexural strength and deflection, average safety rates of 22% and 34% were achieved, respectively.

Table 9 shows the evaluation results for the cases where the strengthening limit coefficient was applied during the service load stage. For flexural strength, it was observed that the evaluated value achieved an average safety factor of 12% than experimental value, and the deflection was evaluated to be approximately 23% smaller than the experimental value; thus, a sufficiently conservative evaluation was performed. The flexural strength evaluation results of specimens ARLo2 and CaLo1S were still found to be larger than the experimental results. In specimen ARLo2, the bond area between the textile and matrix was significantly reduced during the textile arrangement process, and CaLo1S also faced the problem of slippage occurring between the textile and matrix because of the tensile force to straighten the carbon fiber [31,34]. Therefore, it is believed that a sufficient safety factor can be achieved by supplementing the problems when applying the TRM.

## 5. Conclusions

In this study, a conservative evaluation and design method for TRM beams was proposed assuming a strengthening limit reflecting the uncertainty of the TRM reinforcement and considering the results of previous studies.

1. The TRM beams exhibited sufficient efficiency in the service load and yield stages. After the steel reinforcement yielded, the textile was damaged, slipped, and intermediate crack debonding occurred; thus, it was not sufficiently resistant to the load. Therefore, the point at which the steel reinforcement of the TRM beam yielded was defined as the TRM strengthening limit.;
2. Based on the TRM strengthening limit, 0.81 was suggested as the coefficient for flexural strength. The coefficient of 0.66 for the deflection was calculated to be applied to the effective moment of inertia.;
3. Conservative evaluation was possible when the suggested strengthening limit coefficient was applied to both the TRM flexural strength and deflection evaluations. Therefore, it is expected that conservative designing is possible if the strengthening limit coefficient is applied when designing the TRM beam.

This study considered a small number of specimens with premature failure, and thus, an increased accuracy needs to be achieved in the future through a continuous increase in the number of specimens. In particular, to reflect the characteristics of each textile, it is necessary to continuously expand the data for PBO, AR-glass, carbon, etc, respectively.

## Figures and Tables

**Table 1:** Detailed specification of the AR-glass and carbon textiles.

Properties and Geometric Parameters	AR-Glass Textile	Carbon Textile
Tensile strength of filament (MPa)	1789	4900
Modulus of elasticity of filament (GPa)	68	230
Elongation of filament	0.0262	0.022
Number of filaments per roving	1600	12,000
Area per one layer (textile) (mm^2^)	2.952	2.772

**Table 2:** Average compressive strength of concrete and polymer mortar.

Matrix (MPa)	No. 1	No. 2	No. 3	Average
Concrete	43.72	44	38.85	42.19
Polymer mortar	49.42	48.95	47.33	48.57

**Table 3:** Flexural and bond strength of polymer mortar.

Property	Strength (MPa)	Standard of KS (MPa)
Flexural	8	More than 6
Bond	1.5 (with primer 1.8)	More than 1

**Table 4:** Detailed specification of TRM beam.

Specimen	Textile Configuration and Reinforcement Amount			Straightening Force
	Lamination	Layer	Reinforced Ratio	
RC	-	-	-	-
ARLo1	3	1	20.5%	-
ARLo1S	3	1		792 N
ARLo2	3	3	61.5%	-
CaLo1	1	1	21.59%	-
CaLo1S	1	1		679 N
CaLo2	3	1	64.9	-
CaLo2S	3	1		2037 N
CaO	2	3	129.6%	-

**Table 5:** Summary of failure modes.

Specimen	Failure Mode ^1^
RC, CaLo2	C
ARLo1	R+IC D, C
ARLo2	R, C
CaLo1	IC D, R, C
ARLo1S, CaLo1S, CaLo2S, CaO	IC D, C

^1^ C, R and IC D denote concrete crushing, textile rupture and intermediate crack debonding of the TRM, respectively.

**Table 6:** Experimental results of load and deflection.

Specimen	Uncracked		Service		Yield		Ultimate	
	Load (kN)	Def. (mm)	Load (kN)	Def. (mm)	Load (kN)	Def. (mm)	Load (kN)	Def. (mm)
RC	5.68	0.75	22.94	4.6	29.59	6.02	31.81	21.94
ARLo1	6.91	0.77	23.8	4.24	35.51	6.96	-	-
ARLo1S	-	-	32.05	4.49	38.46	5.9	39.45	13.36
ARLo2	1.72	0.29	21.33	4.27	35.26	7.36	-	-
CaLo1	3.82	0.55	23.79	4.6	36.49	8.05	38.22	20.78
CaLo1S	4.19	0.46	19.85	4.11	34.4	6.71	-	-
CaLo2	2.47	0.09	26.75	3.87	40.44	6.97	43.04	15.11
CaLo2S	-	-	28.36	4.67	42.66	7.76	-	-
CaO	-	-	25.52	4.8	41.92	9.14	43.15	12

**Table 7:** Comparison between experimental and predicted results.

Beam	Uncracked						Service						Yield					
	$P_{exp}$	$P_{prel}$	$\frac{P_{exp}}{P_{pre}}$	$\delta_{exp}$	$\delta_{pre}$	$\frac{\delta_{exp}}{\delta_{pre}}$	$P_{exp}$	$P_{pre}$	$\frac{P_{exp}}{P_{pre}}$	$\delta_{exp}$	$\delta_{pre}$	$\frac{\delta_{exp}}{\delta_{pre}}$	$P_{exp}$	$P_{pre}$	$\frac{P_{exp}}{P_{pre}}$	$\delta_{exp}$	$\delta_{pre}$	$\frac{\delta_{exp}}{\delta_{pre}}$
ARLo1	6.91	8.27	0.84	0.77	0.27	2.85	23.8	27.24	0.87	4.24	3.89	1.09	35.51	40.13	0.88	6.96	6.35	1.1
ARLo1S	-	8.62	-	-	0.28	-	32.05	27.33	1.17	4.49	3.84	1.17	38.46	40.58	0.95	5.9	6.36	0.93
ARLo2	1.72	8.27	0.21	0.29	0.27	1.07	21.33	27.96	0.76	4.27	3.78	1.13	35.26	42.8	0.82	7.36	6.43	1.14
CaLo1	3.82	8.27	0.46	0.55	0.27	2.04	23.79	26.93	0.88	4.6	3.82	1.2	36.49	40.22	0.91	8.05	6.35	1.27
CaLo1S	4.19	8.58	0.49	0.46	0.28	1.64	19.85	27.33	0.73	4.11	3.84	1.07	34.4	40.62	0.85	6.71	6.36	1.06
CaLo2	2.47	8.27	0.3	0.09	0.27	0.33	26.75	28.09	0.95	3.87	3.79	1.02	40.44	43.02	0.94	6.97	6.43	1.08
CaLo2S	-	9.16	-	-	0.3	-	28.36	28.09	0.99	4.67	3.71	1.26	42.66	44.18	0.97	7.76	6.47	1.2
CaO	-	8.27	-	-	0.27	-	25.52	28.84	0.88	4.8	3.59	1.34	41.92	47.29	0.89	9.14	6.57	1.39
Mean	-		0.57	-		1.59	-		0.91	-		1.16	-		0.9	-		1.15
S.D			0.32			0.96			0.13			0.1			0.05			0.13
Variance			0.1			0.913			0.017			0.01			0.002			0.017

**Table 8:** Failure mode, flexural strength, and deflection of each reference.

Reference	Beam	Textile Type	Failure Mode	Flexural Strength			Deflection		
				$M_{y,exp}$ (kN·m)	$M_{y,pre}$ (kN·m)	$\frac{M_{y,exp}}{M_{y,pre}}$	$\delta_{y,exp}$ (mm)	$\delta_{y,pre}$ (mm)	$\frac{\delta_{y,exp}}{\delta_{y,pre}}$
[21]	S1-T1-P1-1	PBO	C	36.05	34.05	1.06	17.34	15.28	1.13
	S1-T1-P1-2			36.02	34.05	1.06	17.72	15.28	1.16
	S2-T1-P1			20.26	17.7	1.14	14.65	13.26	1.1
	S2-T1-P2		IC D	22.68	18.97	1.2	15.28	13.42	1.14
	S2-T1-P3-1			23.73	20.25	1.17	15.81	13.59	1.16
	S2-T1-P3-2			20.75	20.25	1.02	21.16	13.59	1.56
	S2-T2-P2			20.22	18.97	1.07	12.47	13.51	0.92
	S2-T2-P3			22.4	20.25	1.11	12.31	13.68	0.9
[13]	S2-T2-P2			20.22	19.33	1.05	12.47	13.16	0.95
	S2-T2-P3			22.4	20.62	1.09	12.31	13.3	0.93
[27]	T1	AR-glass	R	7.57	9.01	0.84	7.48	6.34	1.18
	T1O		IC D, R	8.6	9.01	0.95	8.18	6.34	1.29
	T2		IC D, R	7.85	9.28	0.85	10.02	6.38	1.57
	T3		R	8.71	9.56	0.91	8.53	6.42	1.33
[34]	ARLo1		R+IC D, C	7.99	9.03	0.88	6.96	6.35	1.1
	ARLo1S		IC D, C	8.65	9.13	0.95	5.9	6.36	0.93
	ARLo2		R, C	7.93	9.63	0.82	7.36	6.43	1.14
	CaLo1	Carbon	IC D, R, C	8.21	9.05	0.91	8.05	6.35	1.27
	CaLo1S		IC D, C	7.74	9.14	0.85	6.71	6.36	1.06
	CaLo2		C	9.1	9.68	0.94	6.97	6.43	1.08
	CaLo2S		IC D, C	9.6	9.94	0.97	7.76	6.47	1.2
	CaO		IC D, C	9.43	10.64	0.89	9.14	6.57	1.39
Mean				-	-	0.99	-	-	1.16
S.D.				-	-	0.11	-	-	0.18
Variance				-	-	0.013	-	-	0.034

**Table 9:** Comparison of experimental and evaluated results at the service load stage.

Specimen	$M_{s,exp}$ (kN·m)	$M_{s,eva}$ (kN·m)	$\frac{M_{s,exp}}{M_{s,eva}}$	$\delta_{s,exp}$ (mm)	$\delta_{s,eva}$ (mm)	$\frac{\delta_{s,exp}}{\delta_{s,eva}}$
ARLo1	5.36	4.97	1.08	4.24	5.89	0.72
ARLo1S	7.21	4.98	1.45	4.49	5.82	0.77
ARLo2	4.8	5.09	0.94	4.27	5.73	0.75
CaLo1	5.35	4.91	1.09	4.6	5.79	0.79
CaLo1S	4.47	4.98	0.9	4.11	5.82	0.71
CaLo2	6.02	5.12	1.18	3.87	5.74	0.67
CaLo2S	6.38	5.2	1.23	4.67	5.63	0.83
CaO	5.74	5.26	1.09	4.8	5.44	0.88
Mean	-	-	1.12	-	-	0.77
S.D	-	-	0.16	-	-	0.06
Variance	-	-	0.026	-	-	0.004

## Data Availability

Data is contained within the article.
